# On how to generalize species-specific conceptual schemes to generate a species-independent Conceptual Schema of the Genome

**DOI:** 10.1186/s12859-021-04237-x

**Published:** 2021-09-30

**Authors:** Alberto García S., Juan Carlos Casamayor

**Affiliations:** PROS Research Center, Universitat Politècnica de València, Camino de Vera, Valencia, Spain

**Keywords:** Conceptual modeling, Genomics, Bioinformatics

## Abstract

**Background:**

Understanding the genome, with all of its components and intrinsic relationships, is a great challenge. Conceptual modeling techniques have been used as a means to face this challenge. The heterogeneity and idiosyncrasy of genomic use cases mean that conceptual modeling techniques are used to generate conceptual schemes that focus on too specific scenarios (i.e., they are species-specific conceptual schemes). Our research group developed two different conceptual schemes. The first one is the Conceptual Schema of the Human Genome, which is intended to improve Precision Medicine and genetic diagnosis. The second one is the Conceptual Schema of the Citrus Genome, which is intended to identify the genetic cause of relevant phenotypes in the agri-food field.

**Methods:**

Our two conceptual schemes have been ontologically compared to identify their similarities and differences. Based on this comparison, several changes have been performed in the Conceptual Schema of the Human Genome in order to obtain the first version of a species-independent Conceptual Schema of the Genome. Identifying the different genome information items used in each genomic case study has been essential in achieving our goal. The changes needed to provide an expanded, more generic version of the Conceptual Schema of the Human Genome are analyzed and discussed.

**Results:**

This work presents a new CS called the Conceptual Schema of the Genome that is ready to be adapted to any specific working genome-based context (i.e., species-independent).

**Conclusion:**

The generated Conceptual Schema of the Genome works as a global, generic element from which conceptual views can be created in order to work with any specific species. This first working version can be used in the human use case, in the citrus use case, and, potentially, in more use cases of other species.

## Background

Conceptual modeling (CM) is the activity of describing aspects of the world for the purpose of understanding and communication [1]. Regardless of the research area, it answers fundamental questions by identifying what concepts are relevant and their relationships with each other. Conceptual models make mental representations of the world explicit, which helps to establish common ontological frameworks that facilitate both communication and knowledge evolution in complex domains [2].

An example of such a convoluted and vast domain is genomics, where understanding the genome and all of its intrinsic relationships in order to decipher the code of life is a huge challenge. There are two main reasons for the complexity of the genomic domain. The first one is the existence of relevant concepts that are not clearly defined. Even the definition of the most elemental concepts, like the concept of “gene”, are open to discussion [3]. The second one is that it is an ever-changing domain, with new knowledge emerging continuously [4]. Therefore, the genomic domain is a particularly good candidate for applying CM techniques.

Our research group has extensive experience applying CM techniques in the genomic domain. For years, our main goal has been to understand, identify, and conceptualize those elements that are relevant in this domain. As a result of this work, two different conceptual schemes have been generated. The first one is the Conceptual Schema of the Human Genome (CSHG), which is intended to improve Precision Medicine and genetic diagnosis. The second one is the Conceptual Schema of the Citrus Genome (CSCG), intended to identify the genetic cause of relevant phenotypes in the agri-food field.

The CSHG is proof of how CM can help to improve domain understanding and communication. For years, our main research line has focused on humans, and the CSHG has provided a more explicit and precise understanding of the human genome. The CSHG has proven to be valid and useful, and it has been successfully applied in a series of real-world use cases. Among the most recent works, the following can be highlighted:

- Identifying and managing genomic variations related to the treatment of Alzheimer’s disease [5].
- Developing a CM-based framework to improve the data quality processes of precision medicine [6].
- Reporting of early diagnosis of alcohol sensitivity [7].
- Identifying variations with a relevant role in the development of colorectal cancer [8].
- Developing Genome Information Systems to: (i) improve the diagnosis of congenital cataracts [9], (ii) support the prioritization of variations [10], (iii) support the variation annotation process [11], and (iv) increase interaction and collaboration in the process of diagnosing genetic diseases [12].

In parallel with the above research, we have carried out additional work in a special use case in a new and different context in which the subject under study is not humans but citrus. A new Conceptual Schema (CS) has been developed for the citrus genome with the collaboration of the Valencian Institute of Agrarian Research (IVIA). This CS serves the IVIA as ontological support to better perform their research regarding the genetic improvement of crops. The workflow developed is composed of the following steps: *Step 1*: plant genome sequencing is performed; *Step 2*: variations of interest are identified; *Step 3*: genes of interest to improve a desired citrus trait (e.g., drought resistance) are identified; *Step 4*: genetic modification techniques are applied to citrus crops [13].

The genome is what explains what we humans understand by life on our planet. Since we share a common conceptual background, genome representation is a problem that affects all species of living beings. However, having a CS that only focuses on the human genome can be seen as a limitation in this context. A study of any different species could require a new CS to be created, or adapted, in order to appropriately cover its particularities. Our research group has developed two different conceptual schemes: the CSHG and the CSCG. However, their conceptual background is assumed to be the same because in both cases we are talking about the genome.

### Conceptual Schema of the Human Genome

For years, the creation of a CSHG has been the main goal and a fundamental tool of our work [14]. The result is a CS that is divided into multiple views, which has provided us with a fundamental tool to communicate more effectively with domain experts and to develop Model-Driven Development Genome Information System. As more knowledge about the genomic fundamentals of life has accumulated, the CSHG has evolved in parallel. The CSHG has had two major updates.

Version 1 of the CSHG was the first attempt to generate a holistic conceptualization of the genomic domain. This version precisely defined the most basic concepts of the domain, ignoring some of the more complex aspects. (e.g., pseudo-genes or proteins coded by multiple genes). Version 1 focused on characterizing genes, their mutations, and their phenotypic aspects. Version 1 was divided into three views: Gene-Mutation, Genome, and Transcription. The Gene-Mutation view modeled the concept of gene. It characterized its fundamental parts, including gene variations (e.g., insertions or deletions), regulatory elements (for instance, promoters or terminators, and sequence parts (e.g., coding regions). The Genome view modeled individual genomes. This view offered a general perspective of the genome, structuring genomes in chromosomes, and structuring chromosomes in segments. Chromosome segments were divided into genic segments and non-genic segments. The transcription view modeled the protein-coding process. This view included primary transcripts, exons, introns, spliced transcripts, open reading frames, and proteins.

The first major update of the CSHG (from version 1 to version 1.1) included the Phenotype view in the model. Version 1.1 provided a more consistent CS. Syndromes (pathologic phenotypes) were included in the model with a multi-level classification based on their severity and characteristics. Genotype information was linked to phenotype information, in order to better model the effects of variations in the genome. The Phenotype view is linked to variations to indicate whether a variation is responsible for modifying a phenotypic aspect.

The second major update of the CSHG (from version 1.1 to version 2), changed how the genome sequence is comprehended and represented. This version changes the perspective from gene-focused to chromosome-focused. The concept of gene is no longer the main element of the genome. The reason for this is that it is not always feasible to describe the DNA structure in terms of genes. The main element is the *chromosome element*. This change allows any relevant part of the genome (not just genes) to be easily represented and characterized. Three more changes were made to the CSHG. The first change is that the Genome view was removed from Version 2 because the genome of each individual human should be clearly differentiated from the generic human genome. This change allows genomic analysis to be performed more easily. The second change is the explicit representation of Single Nucleotide Polymorphisms (SNPs). The third change is the addition of the pathways. Pathways are represented as inter-dependent events where a set of inputs produces a set of outputs.

This last version of the CSHG is divided into five views: (i) the *structural view* describes the structural parts that determine the sequence of the genome; (ii) the *transcription view* models the elements that take part in the protein-coding process; (iii) the *variation view* focuses on the structural changes in the genome sequence; (iv) the *pathway view* breaks down metabolic pathways into their fundamental events, specifying the entities that take part in them; and (v) the *bibliography and datatabank view* provides information regarding the origin of the data.

### Conceptual Schema of the Citrus Genome

Citrus is a particularly relevant crop. It is cultivated worldwide with a production of more than 100 million tonnes. The Citrus genus includes oranges, lemons, grapefruits, and pummelos, among others. In total, it is composed of more than 1600 species. Citrus genome resources are abundant. The first citrus variety was sequenced in 2003 [15] and several genomes have been sequenced since then. By the time this article was written, more than 67 species have been sequenced multiple times, with more than 200,000 genes identified (https://www.citrusgenomedb.org/data_overview/1). Comparative genomics is composed of a broad set of analyses, including variations of gene content, large genome rearrangements, structural variants, or small polymorphisms. Our use case focuses on studying small polymorphisms, more specifically, single nucleotide polymorphisms (SNPs) and small insertions and deletions (INDELs). These variations are of great interest for plant breeding. They have proven to be critical determinants for major traits of agricultural interest.

Unlike the CSHG, which was developed to be as generic as possible in order to serve multiple use cases, the CSCG was developed for a specific use case. Because of that, the modeling process and the philosophy of the resulting CS are notably different. The use case that motivated the generation of the CSCG consists of establishing reliable genotype-phenotype relationships, i.e., the observable traits in the varieties (phenotype) that are caused by the genetic code (genotype). An example is the variations in the genetic code that make a variety drought resistant. This is a significantly different type of study compared to the ones that we worked on before when working with the human genome. In the case of the human genome, the studies focus on identifying relevant variations (i.e., variations that are known to cause a given condition) in populations, especially for clinical purposes in precision medicine, where early diagnosis and selection of the right treatment become the main goals. In the case of the citrus genome, experts focus on identifying which variations are relevant (e.g., which variations cause a given condition).

In citrus studies, it is crucial to properly prioritize (e.g., identify and select) those variations that have an impact on the phenotype, specifically focusing on those variations that could have a notorious impact on a trait of interest of citrus plants. The fact is that these analyses are problematic, inefficient, and involves several manual tasks that are slow and difficult to perform, and are prone to human errors. We have grouped these tasks in a four-step workflow:

- Step 1: Plant genome sequencing. The genome sequence of citrus plants of interest is obtained and compared to a reference sequence. A set of identified variations are associated with each sequenced citrus variety. It is worth mentioning that several crops from the same species that have slightly different characteristics are sequenced.
- Step 2: Identification of variations of interest. The variations that could have a potential link with phenotypes of interest are identified through orthology prediction and statistical methods. The identification process is divided into three tasks:
  1. Select Variety Groups: There are tens of sequenced citrus varieties. Working with multiple varieties is a hard task because of the huge amount of data that each one has associated with it. In order to work with such a huge amount of data, bioinformaticians need to work with a subset of the data. The selection of this subset is done based on specific phenotypes of interest. Consequently, two groups are created in this task. The first group is composed of a set of citrus varieties that highly express a phenotype of interest. The second group is composed of a set of citrus varieties that do not express it. Examples of phenotypes of interest include fruit sweetness, resistance to drought, or the absence of premature fruit abscission.
  2. Compare Groups: The next task is to compare the two groups. There are a plethora of attributes and variables that can be used to filter the data prior to the comparison exercise. This step is crucial for two reasons. The first reason is to remove low-quality data and reduce noise. The second reason is to reduce the amount of genomic data in order to speed up the comparison. Examples of such filtering operations include establishing thresholds for data attributes such as the read depth or delimiting the region of the genome to be analyzed. Although comparing two citrus varieties or applying a single filter are challenging but feasible tasks, as the number of varieties in the defined groups, or the number of applied filter increase, the cost and complexity of these tasks increase dramatically. Even though comparing two citrus varieties or applying a single filter are feasible tasks, as more varieties or filters are included, the cost and complexity of these tasks increase dramatically.
  3. Visualize Result: The amount of identified variations can be unmanageable after comparing the groups. Users need to *examine* the data fluidly to identify potential genetic variations of interest. By *examine*, we mean visualize how the data is distributed based on specific criteria and interactively analyze it (e.g., showing or hiding data columns and performing data operations such as grouping, sorting, pivoting).
- Step 3: Characterization of genes of interest. Genes of the sequenced citrus varieties that have their expression, efficiency, or functionality modified in a disruptive way by variations of interest are identified and analyzed. As a result, assumptions regarding potential genes of interest that require experimental validation emerge. Genes of interest are those that have a significant role in a phenotype of interest.
- Step 4: Application of genetic modifications. The previously obtained assumptions are validated by applying genetic modifications through molecular techniques.

The generated knowledge is highly valuable to researchers because it allows citrus varieties to be modified so that they can potentially increase or decrease the level of expression of phenotypes of interest. However, as a consequence of the complexity of these tasks, extracting knowledge is slow, time consuming, and complex and requires considerable effort.

An additional aspect is to study the implications of relevant variations in citrus varieties at an evolutionary level. The origin of citrus has been a matter of controversy [15]. Nevertheless, the phylogeny of ancestral species and their relationship with domesticated varieties have been determined using genomic, phylogenetic, and biogeographic analyses [16]. The findings of Wu et. al. indicate that evolutionary relationships between species of the same genus should be taken into account, which raises the need for conceptualizing their underlying mechanisms. Our approach is the first one that ontologically defines these aspects in a CS. These relationships are modeled by describing the orthology group concept, which allows us to infer relationships between citrus species in both genes and proteins.

When working with citrus domain experts, we noticed that they work more with technologically-oriented data rather than purely biological data. For instance, they rely on the use of variant annotations and functional effect prediction software. This data mixes biological and non-biological information, being much more format-file oriented. This means that citrus data is stored as obtained, which results in the mix of different concepts. In our previous work with human genomic data, the genomic data that we accessed was transformed by theirs maintainers into a specific model, increasing its abstraction and making it technological-agnostic; but the citrus data that we worked with did not undergo this process. Thus, the information is tied to the technologies used and their limitations. For example, there is no distinction between qualitative data that indicates the quality of the sequencing process of variants and their biological significance. Consequently, there is a loss of the semantics that limits domain understanding.

We are perfectly aware that, generally speaking, the genome provides the common, holistic knowledge to understand life as we perceive it on our planet, independently of any particular species. Nevertheless, our experience in the real working domains of human genome-based applications (e.g., precision medicine) in the CSHG case, and our experience in the case of analyzing links between genome variations and their associated phenotypes in the CSCG case have clearly shown us that the conceptual views that are used in these different working environments are different. Depending on the peculiarities of the problem under investigation, the relevant data that must be considered changes.

To deal with these particularities in the case of citrus, the CSCG was developed following a conceptual modeling method that emphasizes explicitly separating biological and non-biological data by adopting a multi-model-oriented approach. It proposes starting with a purely-biological CS to which additional non-biological conceptual schemes are appended. The resulting CS takes into account the intricate relationships between these two types of data, allowing us to recover the previously hidden semantics of the data. A full view of the CSCG can be seen in [17].

Even though the scenarios that have motivated the generation of these conceptual schemes are different in their particularities, they do share common concepts. This led us to the question of whether each species actually need a CS that is adapted to it specifically, or if it is possible to have a single, holistic CS that works for every species and that can adapt to the idiosyncrasies of individual species.

Our work has been limited to the particularities of the selected working domains (the human genome and the citrus genome), where different genome components are considered to be relevant depending on the purpose of the corresponding data analytics. Nevertheless, it seems clear that the inner workings of the eukaryotic genome share the same underlying foundations (i.e., the genome of a eukaryotic cell consists of a set of chromosomes located in the nucleus with extrachromosomal DNA found in the mitochondria) [18]. For example, the spatial arrangement of eukaryotic species shares the same strategy, i.e., linear chromosomes [18]. Besides, centromeres and telomeres are composed of tandem arrays of repetitive sequence in eukaryotic cells [19] and, when compared to prokaryotic cells, eukaryotic cells has led to more complex and versatile regulatory strategies of DNA replication [20]. Also, gene orthology studies show that there are genes with similar functionality among species. In addition, low-level interactions of biological pathways (i.e., interactions among molecules in a cell leading to a specific product or cell change) change very little between closely related species.

In this work, we present a new CS, called the Conceptual Schema of the Genome (CSG), that is species-independent. The CSG provides a holistic perspective of the genome so that any specific working domain could have its conceptual view inferred from that global CS. The CSG is based on the two previously existing ones (i.e., the CSHG and the CSCG). It not only generates conceptual views to work in the human domain and the citrus domain, it also potentially works with any eukaryote species.

## Methods

This article describes the changes made in the CSHG in order to obtain the first version of the CSG. To do this, the CSHG is compared with the CSCG to identify their similarities and differences. The comparison is performed on a ordered per-view basis:

1. The Structural view.
2. The Transcription view.
3. The Variation view.
4. The Pathway view.
5. The Bibliography and Databank view.

The changes needed to provide an expanded, more generic version of the CSHG are analyzed and reported. We have designed a holistic CSG that is ready to be adapted to any specific working genome-based context (corresponding to studies that affect different species of what we mean by life on our planet). The identification of the different pieces of genome information used in different genomic case studies has been essential in achieving our goal.

The experience accumulated with the analysis of both human genome data and citrus genome data has lead us to designing a unified conceptual schema of the genome that is intended to capture the essentials of the genome structure by identifying all of the relevant conceptual concepts that could represent the holistic knowledge associated to the genome regardless of the species. We envision this Conceptual Schema of the Genome (CSG) as a holistic artifact that provides a common conceptual background that could be used with any particular species by creating the conceptual view that satisfies the needs of any working domains (such as the ones supported by the CSHG and the CSCG presented in this paper). The CSG can be seen as a universal conceptualization of the relevant genome properties that is not exclusively linked to any particular species and that has the capability of providing the required conceptual view to be applied to any genome-based study.

After obtaining the CSG, it is compared to the other two conceptual schemes in the Discussion section in order to validate its correctness and completeness.

## Results

Having a CSG that could be used for any species could open new horizons. We have already identified and detailed our two existing conceptual schemes: the CSHG to work with humans and the CSCG to work with citrus. Since the CSHG is the most mature of the two, it has been used as a basis to identify which parts need to be improved (i.e., generalized or specialized) in order to transform it into a potentially species-independent CSG. To do this, we compared the CSHG with the CSCG, and we have reported the findings (since preserving the view structure facilitates the management of the diversity and complexity of the data involved). Additional considerations are also addressed in this section.

### Structural view

The CSHG offers an abstraction mechanism that allows modeling any existing element contained in the genome sequence, such as genes or intergenic regions. This approach is the same as the one used in the CSCG since the *chromosome element* concept of the CSHG corresponds to the *sequence part* concept of the CSCG. This approach allows us to include any eventual species-specific genomic element. It is an approach that is generic enough to achieve our goal, and it does not require any changes. However, we have identified a significant difference: the CSHG does not have the concept of scaffold.

It is important to note two points regarding the concept of scaffold in the CSHG. The first point is that the existence of this concept is the result of the limitations of current sequencing technologies. Real-life sequencing is far from perfect, and it is not possible to correctly obtain the whole genome sequence at once. Sequencing machines break the genome sequence into many smaller sequences that are read multiple times and then joined. The result is a genome sequence that has gaps of known length. The second point is that every species has scaffolds that compose its genome sequence. This concept is an example of how core concept definitions can be fuzzy and hard to model even among geneticists. The scaffold concept is a conflicting one; some define it as the sequence of a chromosome while others define it as a part of the chromosome with gaps of known length.

**Fig. 1:**
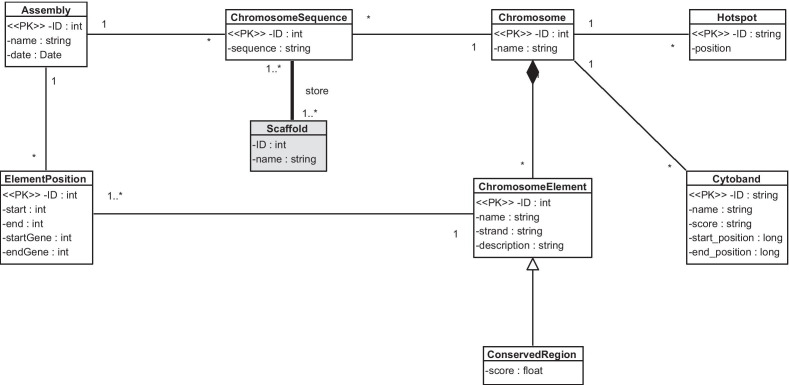
Structural view. The initial CS is depicted in white boxes and the additions are shaded in gray. One class was added: the Scaffold one

Consequently, the concept of scaffold has been incorporated into the new CSG. We represented it as an entity where chromosome sequences are stored. Figure 1 shows the result, where the initial CS is depicted in white boxes and the additions are shaded in gray. This approach allows us to represent scaffolds containing entire chromosome sequences and scaffolds containing just a part of the entire chromosome sequence. This addition is relevant because it allows specific scaffold sequences to be analyzed, which is an important task in some genomic domains [21].

### Transcription view

When the genomic components that structure the Transcription view of both the CSHG and the CSCG are compared, we conclude that they share a high degree of similarity. Chromosome elements are classified into regulatory elements if they regulate the transcription process (e.g., increasing the rate of transcription or stopping it), or into transcriptable elements if they are transcribed into the transcripts that will generate the protein. Since the gene is such a convoluted and complex entity [3, 22, 23], we represent it as a combination of chromosome elements so that the wide range of possible cases can be properly represented. Our representation is in line with what Gerstein et. al. consider the definition of gene, as they state that “a gene is a union of genomic sequences encoding a coherent set of potentially overlapping functional products” [24]. By representing the concepts of gene and transcription this way, we can represent the classical definition of the gene; genes whose sequence contains regulatory elements of other genes; nested genes [25]; or the generation of transcripts from different genes through trans-splicing, which is a phenomenon that has been reported in humans [26]. The identified genomic components are more detailed regarding transcription regulators in the CSHG case because in the citrus case the level of knowledge associated to the study of transcription regulator elements is less strong than in the human case.

However, four elements that exist in the CSCG do not exist in the CSHG and need further clarification. The first element is the concept of intron. The concept of intron was present in the first version of the CSHG, but when geneticists started using the CSHG, we found that this concept was never used, and we decided to remove it. In the case of citrus, intron-located variations are much more relevant and studied.

The second element is the concept of domain. A domain is defined as the basic, independent unit of protein folding, evolution, and function [27]. The CSHG represents proteins as a unique block with a given functionality, without representing that they are composed of multiple smaller, interconnected parts. Domains are included in the CSCG because they are compared in order to infer the evolutionary closeness of the citrus species.

The third element is the concept of the mRNA. The CSHG models the transcription process so that genes produce transcripts, with *protein-coding* being a type of transcript that produces proteins. This way, only those transcripts that are *protein-coding* produce proteins, and transcripts that do not produce proteins can exist. The CSCG models the transcription so that genes produce mRNAs and mRNAs produce proteins. Consequently, those transcripts that do not produce proteins cannot be modeled. The *protein-coding* concept in the CSHG is ontologically equivalent to the mRNA concept of the CSCG. We determined that this concept should be renamed to mRNA in order to make its representation in the CSHG explicit. Besides, the CSCG also defines the structure of the mRNA because the variations located in these regions are studied specifically in this domain. An mRNA is composed of three elements: the 5’ untranslated region (5’ UTR), the coding sequence (CDS), and the 3’ untranslated region (3’ UTR). On the one hand, the CSHG is a more generalizable solution because it allows us to model transcripts that do not produce proteins. On the other hand, the CSCG models the concept of mRNA in a more detailed way because it specifies the structural parts that compose it.

The fourth element is the concept of ortholog group. An ortholog group is defined as a set of genes that are assumed to have evolved from a single gene in a common ancestral species. Contrary to paralogue groups, which are genes created by duplication events, ortholog groups are created by speciation events. The CSCG models the concept of the ortholog group as a collection of genes that have evolutionary correlations and, optionally, a set of the enzymes coded by these genes. For example, an ortholog group can contain a set of genes from three different citrus varieties and the enzyme that these genes produce.

**Fig. 2:**
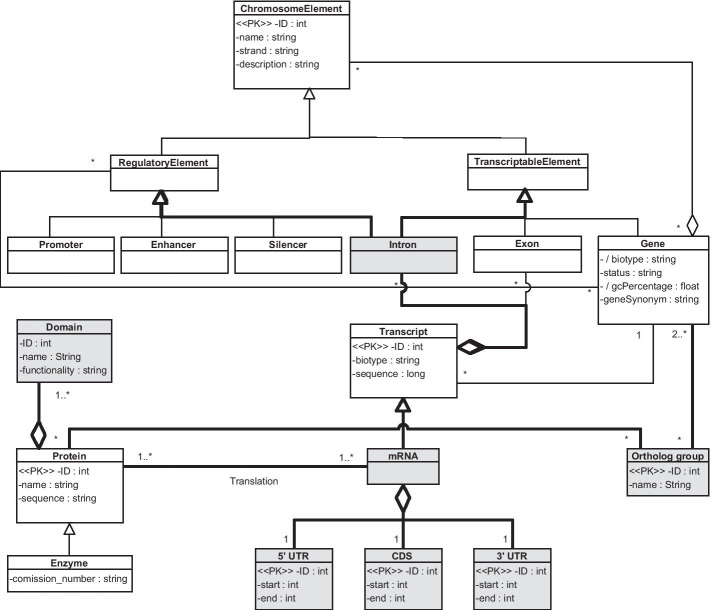
Transcription view. The initial CS is depicted in white boxes and the additions are shaded in gray. Seven classes were added: the intron, as a chromosome element that can be transcriptable or regulatory; the domain, representing the independent building blocks that compose proteins; the ortholog group, to link genes that have evolved from a single gene; and the mRNA and its structural parts: the 5’ UTR, CDS, and the 3’ UTR. Please note that the *ChromosomeElement* class pertains to the structural view, but we have included it to provide additional context

Consequently, in the global CSG, we have included these four elements (see Fig. 2, where the initial CS is depicted in white boxes, the additions are shaded in gray). In order to perform an analysis that specifically studies structural changes in introns [28], we represented this concept in the CSG as a chromosome element that is both regulatory and also transcriptable. It is regulatory since introns can significantly affect gene expression [29, 30]; it is transcriptable because intron-retention (IR) is a common event in some human gene families [31], and IR is gaining attention in the treatment of diseases [32].

We included in the CSG the domain element because decomposing each protein into modular domains is a basic prerequisite for accurate functional classification of biological molecules [33]. The domain is represented as an entity that forms proteins. Therefore, a protein is defined as a set of at least one domain.

We included in the CSG the mRNA element with its structural parts (the 5’ Untranslated Region, the coding sequence (CDS), and the 3’ Untranslated Region) to be able to locate structural changes in these regions and study their consequences [34]. Some transcripts are not processed to yield mRNA; thus, the concept of mRNA is represented as a type of transcript, together with its structural composition (i.e., 5’ UTR, CDS, and 3’ UTR). With this approach, we can represent mRNA, other types of mature RNAs like tRNA, and regulatory RNAs.

We included in the CSG the ortholog group element since gene identification is fundamental to all aspects of biology [35] and allows domain experts to study hundreds of years of evolution by applying phylogenetic and comparative analysis [36]. To facilitate gene functionality analysis, ortholog groups are represented as an entity that relates at least two different genes and (optionally) a set of proteins.

### Variation view

The CSHG represents variations with a good level of generality, but we identified a limitation after comparing it to how the CSCG represents variations. The CSHG has been used in genomic studies that focus on studying genotype frequencies of SNP variations. As a result, the modeling process of the CSHG prioritizes representing genotype frequencies of SNP variations over other types of variations. In contrast, the citrus use case focuses on studying both the genotype frequency of SNP and INDEL variations. The reason why the study of genotype frequencies of INDEL variations is relevant because their high degree of heterozygosity is used to establish taxonomic relationships [37]. Therefore, the genotype frequencies of INDEL variations cannot be represented in the CSHG. Besides, the CSHG represents variations at two different levels because it focuses on studying variations in populations. The first level is the general one, in which general information regarding the variation is represented. The second level is the population one, in which frequencies and other characteristics of a variation in a population are represented. In contrast, the citrus use case focuses on studying variations in individual citruses, and it represents variations in two different levels. The first level is the general one, like in the case of the CSHG. The second level is the individual one, in which frequencies and other characteristics of a variation in a specific individual citrus are represented.

**Fig. 3:**
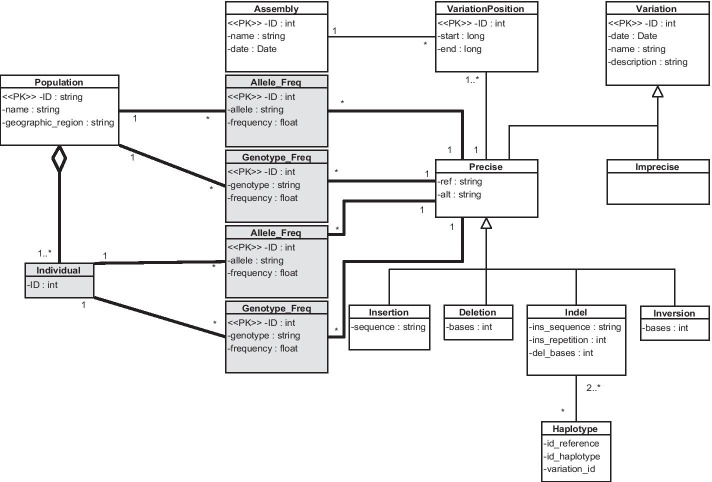
Variation view. The initial CS is depicted in white boxes and the additions are shaded in gray. Five classes have been added: the first one is the concept of individual as a part of a population. The remainder classes indicate genotype and allele frequencies of populations and individuals

To solve this limitation, in the CSG, we generalized the concept of genotype frequency from only being a property of SNP variations, to being a property of every type of variation. With this change, every type of variation has a genotype frequency represented. Additionally, the CSG represents variations in three levels: the general level, the population level, and the individual level. The final result is shown in Fig. 3, where the initial CS is depicted in white boxes and the additions are shaded in gray). Now, individuals are the entities that conform populations, and the CSG represents the frequency of alleles and genotypes in both populations and individuals.

As a side note, this exercise also allowed us to discover elements that are relevant in the citrus domain but that were not initially considered because they are not studied in the working use case. An example is the concept of haplotype, which is defined as a set of variations that statistically appear together [38]. The identification of haplotypes in citrus is an important topic that is being studied [39] and should be included in the CSCG for future studies.

### Pathway view

The CSHG has a highly generic and flexible representation of pathways and their inner processes. It represents the specific events that occur in each pathway, how they are related, and the biological entities that take part in them. The CSCG represents entire pathways as indivisible blocks, ignoring their internal processes. Instead of allowing any biological entity, the CSCG only specifies what enzymes take part in a pathway, and only at a general level rather than at a pathway-specific process level. We conclude that, in this case, no changes in this view are required.

**Fig. 4:**
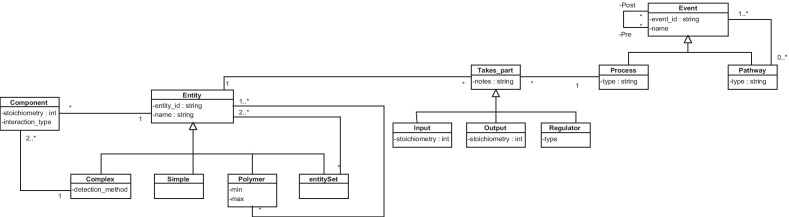
Pathway view. No additions were needed

To illustrate this representation, the pathway view is briefly explained below. Figure 4 shows the pathway view. It is composed of three main concepts, namely, *entity*, *takes_part*, and *event*. The generic concept of entity is defined as any physical element that takes part in a biological pathway. Entities can be one of the following:

- Simple: an atomic entity such as a protein, a monomer, or water.
- Complex: a non-atomic entity that is composed of other entities of any given type.
- EntitySet: a non-atomic entity that groups entities that act together but remain independent, unlike complex entities.
- Polymer: an entity composed of the repetition of either simple or complex entities.

This representation allows us to define entities with the desired level of granularity (i.e., generic entities or more specific ones) and to establish a hierarchical structure for them. The generic concept of the event represents the combination of an organism’s processes. A pathway is defined as a set of ordered biological events, being either atomic processes or other pathways.

Finally, entities take part in processes as input, output, or regulator. Those entities that take part in processes can be merged to determine the inputs, outputs, and regulators of pathways. We decided to make the “take part” relationship explicit because of its rich semantics. The “takes_part” entity needs to be specialized into other entities, and associations cannot be specialized in UML. These specialized classes have specific attributes that are not shared among the others. Thus, we decided to model it as a class.

### Bibliography and Databank view

The CSHG allows us to represent the bibliography of the represented genomic components and how they are identified in external data sources. The CSCG does not include information regarding the bibliography of the data or its origin. Therefore, no changes in the CSHG are required, and the Bibliography and Databank views of the CSHG and the CSG are the same.

## Discussion

One of the main strengths of the CSCG is how well it integrates technologically-oriented data since the citrus use case relies much more upon this data than the human genomic studies that we have analyzed. The CSCG has been modeled using a methodological approach that explicitly separates technologically-related data into independent conceptual schemes that can be assembled like puzzle pieces depending on the needs of specific use cases. For example, a CS was created to conceptualize Gene Ontology annotations. This new CS is not part of the CSCG, but the Gene Ontology data can be integrated if needed by merging the CSCG and the CS of Gene Ontology. The main benefits of this approach are that data integration is straightforward and automated (an example of the benefits of such automation can be found in [13]), there is a direct mapping between biological and technological-oriented concepts, and the final CS has a more complete way of representing those elements whose significance is biased by technologically-oriented data. In addition to the changes proposed, reformulating the CSHG so that its generation process uses this approach can be useful in gaining the benefits mentioned above.

Table 3 shows the number of concepts of the structural, transcription, variation, pathway, and bibliography views in the CSHG, the CSCG, and the CSG. The transcription and variation views are the ones that have the most changes. On the one hand, switching from the CSHG to the CSG increases the number of identified concepts by 28.26%. This means that now the use case studies that worked with the CSHG can: (i) represent the genomic elements that compose transcripts and mRNA better; (ii) identify the domains that form proteins; (iii) perform evolutionary studies using ortholog groups; and (iv) model genotype frequencies at the population level and at the individual level.

On the other hand, switching from the CSCG to the CSG increases the number of identified concepts by 156.52%. This means that now the use case studies that worked with the CSCG can (i) analyze multiple assemblies; (ii) determine the regulatory elements that play an important role in the transcription process; (iii) represent variations with more detail; (iv) decompose pathways into their atomic processes; and (v) incorporate additional information regarding the bibliography and external references of genomic elements.

A relevant insight of this work is that only twelve concepts are shared between the CSHG (26% of the 46 concepts of the CSHG) and the CSCG (52% of the 23 concepts of the CSCG). Genomic use cases are vast and heterogeneous, and additional efforts are needed to generate new views of the global CSG that are suitable for new use cases. A logical conclusion from this situation is that the CSG is probably going to be reevaluated each time that a new use case that greatly differs from the previous ones is studied (Table 1).

**Table 1:** Conceptual schemes concepts comparison

CS	Structural	Transcription	Variation	Pathway	Bibliography	Total
CSHG (1)	8	10	11	13	4	46
CSCG (2)	4	13	4	2	0	23
Shared by 1 and 2	3	6	1	2	0	12
CSG (3)	9	17	16	13	4	59

**Fig. 5:**
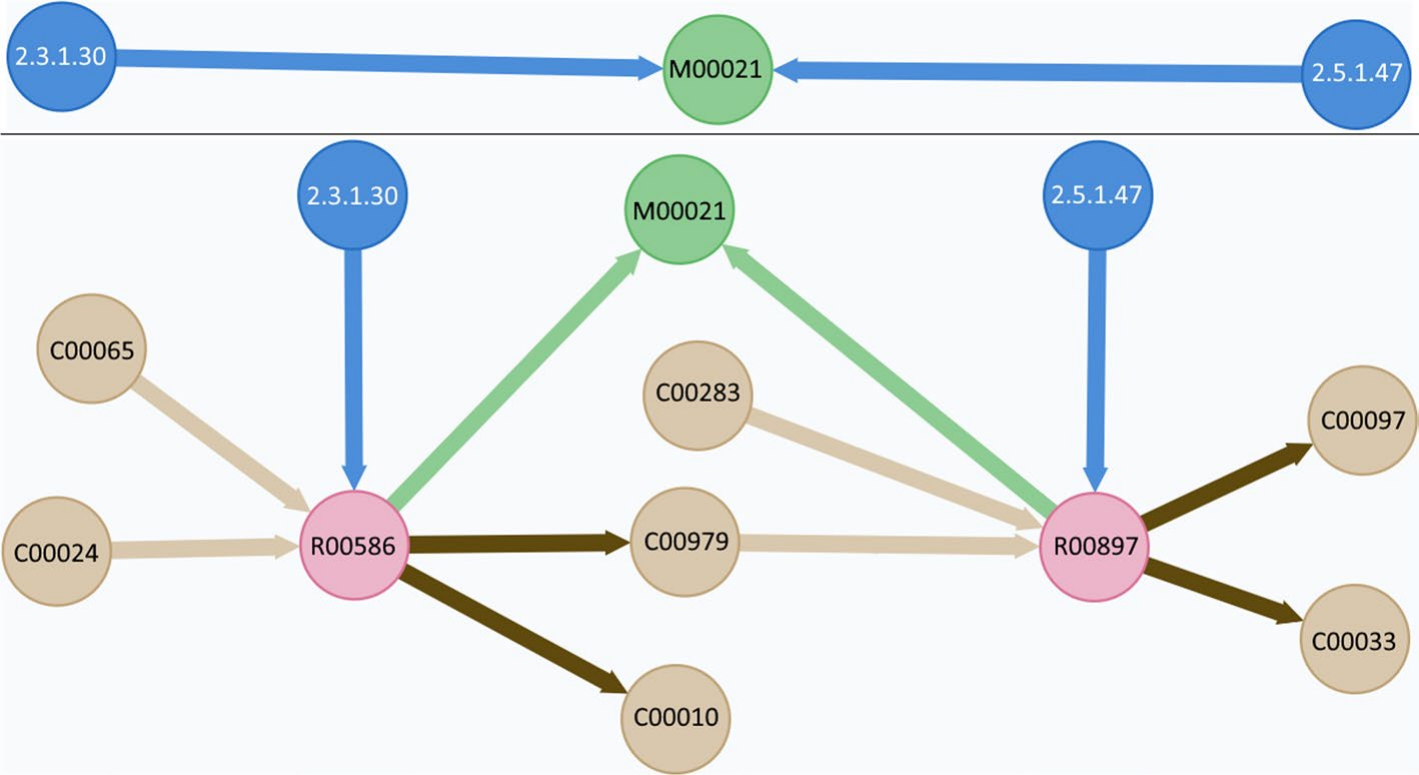
Previous and current representation of the cysteine biosynthesis. Enzymes are represented with blue nodes. Pathways are represented with green nodes. Processes pertaining to pathways are represented with pink nodes. Chemical compounds acting as inputs are represented with light brown nodes and light brown arrows. Chemical compounds acting as inputs are represented with light brown nodes and dark brown arrows

**Fig. 6:**
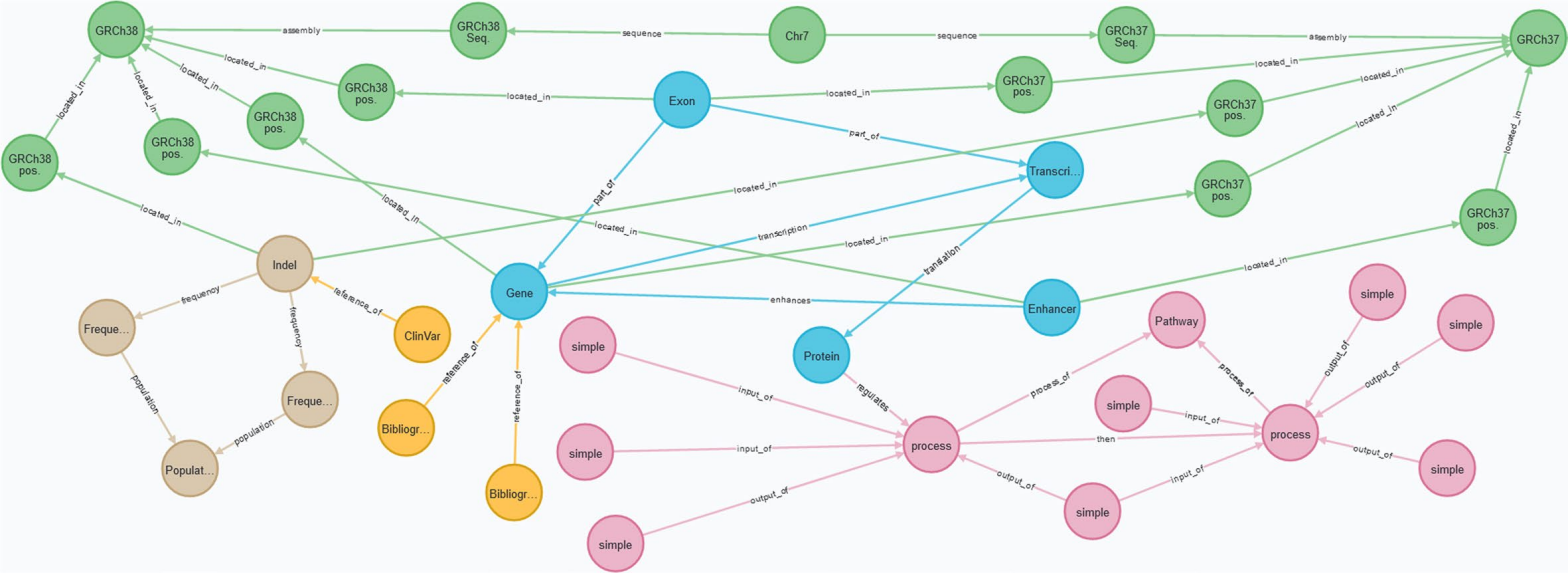
The data gathered for the second example: information related to the NM_000492.3(CFTR):c.57G>A(p.Trp19Ter) variation instantiated with the CSHG. The structural view is represented with green nodes, the transcription view is represented with blue nodes, the variation view is represented with light brown nodes, the bibliography and data bank view is represented with yellow nodes, and the pathway view is represented with pink nodes

**Fig. 7:**
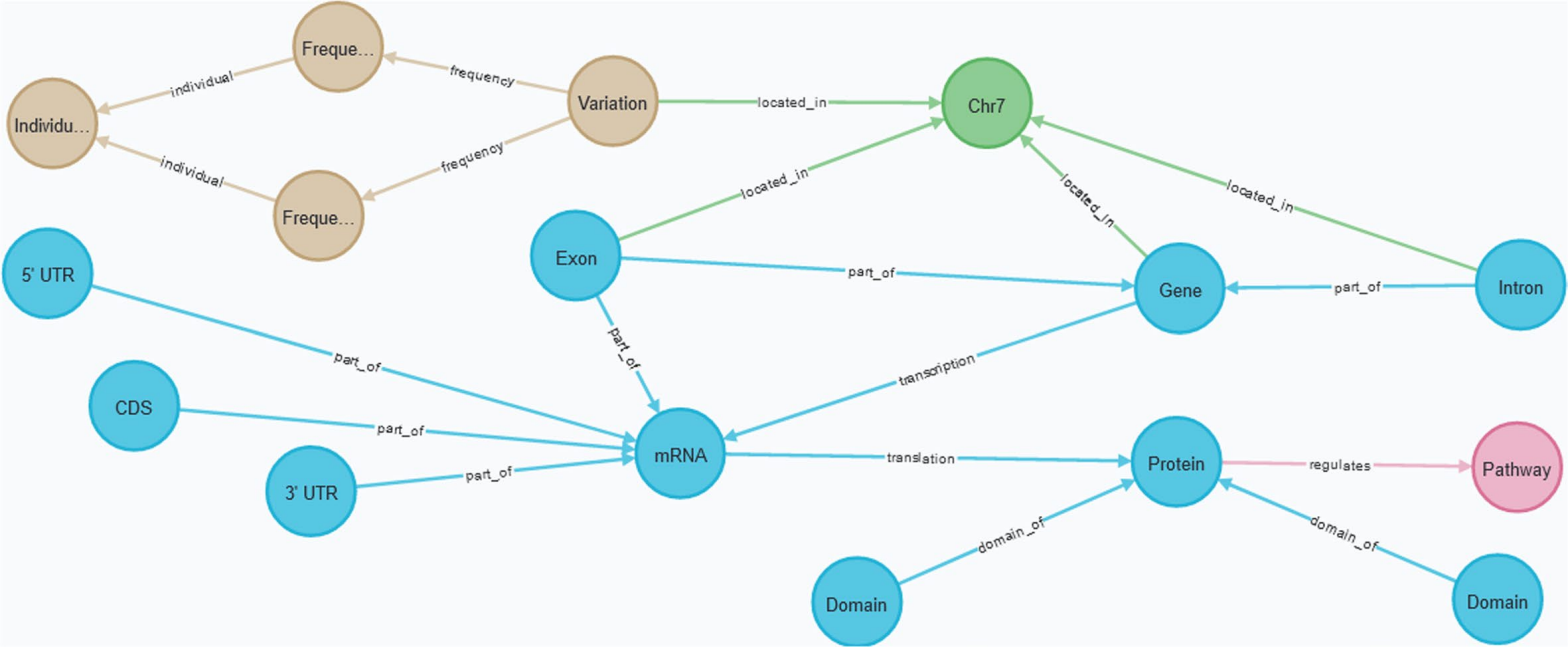
The data gathered for the second example: information related to the NM_000492.3(CFTR):c.57G>A(p.Trp19Ter) variation instantiated with the CSCG. The structural view is represented with green nodes, the transcription view is represented with blue nodes, the variation view is represented with light brown nodes, the bibliography and data bank view is represented with yellow nodes, and the pathway view is represented with pink nodes

**Fig. 8:**
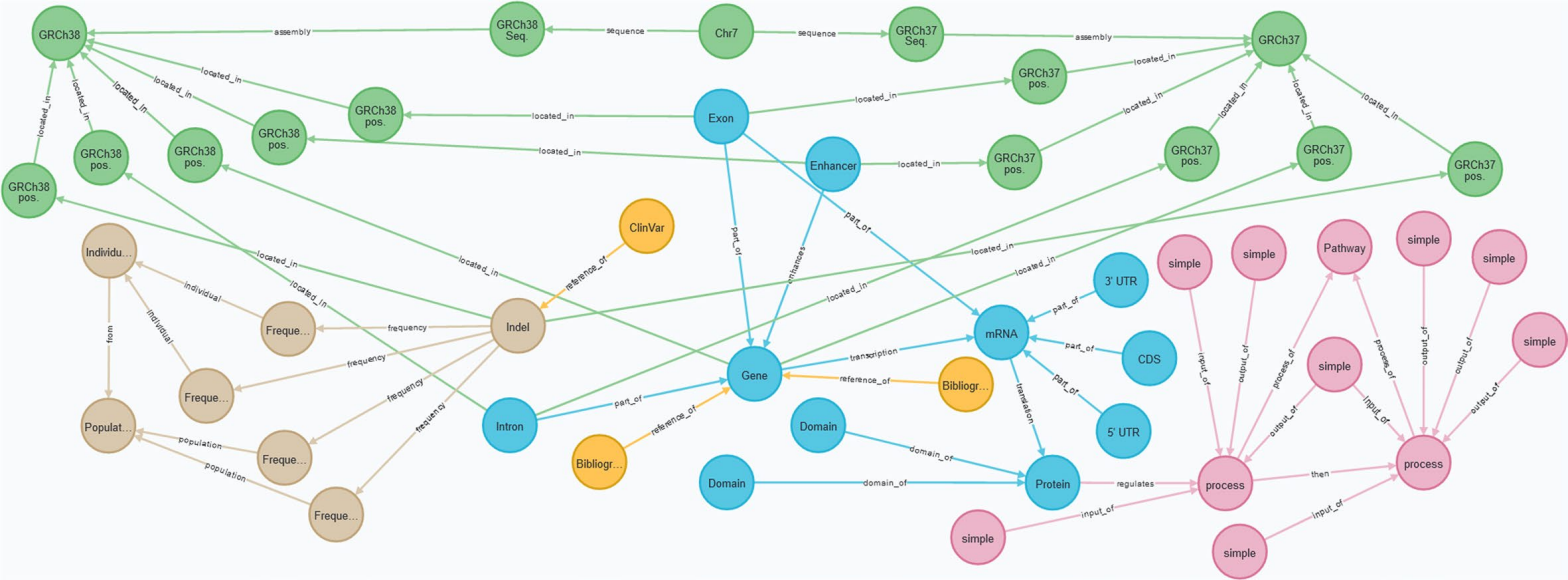
The data gathered for the second example: information related to the NM_000492.3(CFTR):c.57G>A(p.Trp19Ter) variation instantiated with the CSG. The structural view is represented with green nodes, the transcription view is represented with blue nodes, the variation view is represented with light brown nodes, the bibliography and data bank view is represented with yellow nodes, and the pathway view is represented with pink nodes

Let us illustrate the potential benefits of this contribution by discussing two specific examples. First, citrus data is used to show how the CSG provides a better representation of pathways. This example represents the real-world challenge that we have overcome thanks to the reported improvements. Second, human data is instantiated with the three conceptual schemes to compare them. Figs. 5, 6, 7 and 8 illustrate the examples we have just mentioned above using a graph-like representation of the data to ease visualization. The different conceptual schemes in Figs. 1, 2, 3 and 4 are represented using the Unified Model Language (UML), but we instantiated them using Neo4J, a graph-oriented database.

In the first example, we show pathway representation improvements as a consequence of using the CSG. The data has been obtained from the Kyoto Encyclopedia of Genes and Genomes (KEGG) [40]. In the example detailed below, the chemical compounds and processes that take part in the pathway are identified with the KEGG id (e.g., L-Serine is identified with C00065). The enzymes are identified using the Enzyme Commission Number (EC), created by the Nomenclature Committee of the International Union of Biochemistry and Molecular Biology (NC-IUBMB) (https://www.qmul.ac.uk/sbcs/iubmb/enzyme/) [41]. The EC is used by KEGG to identify enzymes that take part in pathways. The Cysteine biosynthesis pathway (M00021) is used as an illustrative example. The previous representation of this pathway showed that M00021 is a concealed process in which two enzymes: serine O-acetyltransferase (2.3.1.30) and cysteine synthase (2.5.1.47) perform an unknown action. The current representation shows that M00021 is a two-step pathway (first, the acetyl-CoA:L-serine O-acetyltransferase proccess, which is identified by the R00586 code; second, the O3-acetyl-L-serine acetate-lyase process, which is identified by the R00897 code), and their inputs and outputs are defined. The first reaction (R00586) converts L-Serine (C00065) and Acetyl-CoA (C00024) into O-Acetyl-L-serine (C00979) and CoA (C00010), and the reaction is catalyzed by the serine O-acetyltransferase enzyme. The second reaction (R00897) converts O-Acetyl-L-serine (C00979) and Hydrogen sulfide (C00283) into L-Cysteine (C00097) and Acetate (C00033) The result is a much deeper knowledge of the inner processes of citrus pathways, which gives researchers more insights. Figure 5 shows both the previous and the current pathway representations. The figure represents a directed graph in which input and output products are shown in light brown nodes, pathways in green nodes, processes in pink nodes, and regulators in blue. Inputs are connected to processes with light brown lines while processes are connected to outputs with dark brown lines.

Before this change, citrus domain experts worked with 330 pathways and 33929 proteins, of which 1273 were enzymes. We updated the information related to pathways using the new approach, resulting in thousands of new elements being created. Table 3 summarizes the new elements obtained (Table 2).

**Table 2:** Newly data created

Entity simple	Takes_part			Event	
	Input	Output	Regulator	Process	Pathway
14,084	64,300	62,417	27,212	25,803	330

The initial 330 pathways were decomposed in 25.803 atomic processes

The enzymes are part of the 14.084 simple entities and these enzymes act as regulators 27.212 times

In the second example, we instantiate the three models (i.e., CSHG, CSCG, and CSG) using data related to a specific variation that is pathogenic to cystic fibrosis in order to examine the limitations of each CS. The variation is identified with the HGVS expression ‘NM_000492.3(CFTR):c.57G>A(p.Trp19Ter)(https://www.ncbi.nlm.nih.gov/clinvar/variation/487391/) [42]. Based on a real-world use case of precision medicine that we are investigating with, the conceptual schemes should be able to model both the variation’s characterization and the following information:

- Structural view: the chromosome where the variation is located.
- Transcription view: the affected genes, their coding sequence, and their regulatory elements; the proteins coded by these genes and their domains.
- Variation view: the variation’s position in the GRCh37 and GRCh38 assemblies, the type of the variation, and the allele frequencies reported in the European population and in a specific individual.

Due to space limitations, we do not show every instantiated element. For instance, the CFTR gene has twenty-seven exons, but we only represent one. Also, since we consider that this working example is a means to discuss the strengths and limitations of each CS, representing the full data set is not our goal.

Figure 6 shows the data using the CSHG, Fig. 7 shows the data using the CSCG, and Fig. 8 shows the data using the CSHG. The structural view is represented with green nodes, the transcription view is represented with blue nodes, the variation view is represented with light brown nodes, the bibliography and data bank view is represented with yellow nodes, and the pathway view is represented with pink nodes. The arcs represent the relationships among nodes. Based on the premises defined above, we interrogated the conceptual schemes with a set of questions, which have been extracted from the use case under study in order to examine their validity and discuss the results. Table 3 shows the questions on a per-view basis.

**Table 3:** Questions to validate the conceptual schemes and results

ID	Question	Results		
		CSHG	CSCG	CSG
*Structural view*				
1	In which chromosome is the variation located?	$\checkmark$	$\checkmark$	$\checkmark$
2	What is the position of the variation in the GRCh37 assembly?	$\checkmark$	$\times$	$\checkmark$
3	What is the position of the variation in the GRCh38 assembly?	$\checkmark$	$\checkmark$	$\checkmark$
Percentages of correct answers		1	0.66	1
*Transcription view*				
4	What gene is affected by the variation?	$\checkmark$	$\checkmark$	$\checkmark$
5	what are the exons of the affected gene?	$\checkmark$	$\checkmark$	$\checkmark$
6	what are the introns of the affected gene?	$\times$	$\checkmark$	$\checkmark$
7	what are the regulatory elements of the affected gene?	$\checkmark$	$\times$	$\checkmark$
8	What proteins are affected by the variation?	$\checkmark$	$\checkmark$	$\checkmark$
9	What domains compose the affected protein?	$\times$	$\checkmark$	$\checkmark$
10	What is the coding sequence of the affected transcript?	$\times$	$\checkmark$	$\checkmark$
11	What is the sequence of the untranslated regions of the affected mRNA?	$\times$	$\checkmark$	$\checkmark$
Percentages of correct answers		0.5	0.875	1
*Variation view*				
12	What is the type of the variation?	$\checkmark$	$\checkmark$	$\checkmark$
13	What are the alleles of the variation?	$\checkmark$	$\checkmark$	$\checkmark$
14	What is the allele frequency of the variation in the European population?	$\checkmark$	$\times$	$\checkmark$
15	What is the allele frequency of the variation in the individual under study?	$\times$	$\checkmark$	$\checkmark$
Percentages of correct answers		0.75	0.75	1
Pathway view				
16	What pathway is regulated by the affected protein?	$\checkmark$	$\checkmark$	$\checkmark$y
17	What specific process of the pathway is affected by the variation?	$\checkmark$	$\times$	$\checkmark$
18	What are the inputs and outputs of the affected process?	$\checkmark$	$\times$	$\checkmark$
Percentages of correct answers		1	0.33	1
*Bibliography and Data Bank view*				
19	What are the external references of the variation	$\checkmark$	$\times$	$\checkmark$
20	What is the bibliography regarding the affected gene?	$\checkmark$	$\times$	$\checkmark$
Percentages of correct answers		1	0	1
Global percentages of correct answers		0.75	0.7	1

As Table 3 shows, the CSG is the most suitable CS for this specific example since it scored 100% of correct answers. Although the global results for the CSHG and the CSCG are similar (70%, 75% respectively), there are significant differences in the per-view results. With regards to the Structural view, the most significant difference is that the CSCG is not able to work with multiple assemblies. There are scenarios, like precision medicine, where it is necessary to work with more than one assembly. However, this approach also increases the complexity of the model because the position of the genomic entities must be extracted from them (i.e., from an attribute to an independent class). In cases where domain users only work with one assembly, the CSCG simplifies the mental model, thus facilitating knowledge discovery.

With regards to the Transcription view, the CSCG outperforms the CSHG despite both having a similar number of entities in this view (10 for the CSHG and 13 for the CSCG). The CSCG focuses on the whole transcription process and its associated elements much more than the CSHG. The CSHG represents the DNA entities in more detail. For example, it provides a characterization based on entity functionality (i.e., regulatory or transcriptable elements), but it lacks depth representing the RNA and amino acid entities (mRNA and proteins). Depending on the working context, it may be worth focusing on the DNA, the RNA, or the amino acid entities.

With regards to the Variation view, the CSHG represents variations at a population level, the CSCG represents variations at an individual level, and the CSG represents both. Once again, the working context plays a decisive role in the development of the underlying CS. The same happens with the Bibliography and the Pathway views: the goals of domain users that motivated the design of the CSCG did not focus on scientific references or the atomic processes of pathways.

We repeated this exercise with the use cases that motivated the generation of the CSHG and the CSCG. As expected, the CSHG outperforms the CSCG in the human case, the CSCG outperforms the CSHG in the citrus case, and the CSG is the most suitable one when comparing the three use cases analyzed. However, this high degree of completeness and adaptability comes at a price. The CSG is bigger and more complex than the others, which reinforces our initial proposal of generating conceptual views from the CSG in order to adapt to the idiosyncrasies of the use cases.

Two conclusions arise from this work: (i) the generated global CSG is correct and complete, and it offers a real and valid solution to our existing use cases; (ii) in order to provide the best solution, conceptual views that deal with the particularities of the specific use cases must be generated.

## Conclusions

Our experience shows that the genomic domain is complex and some of the core concept definitions are fuzzy. Even though the genomic cases that we have worked with share a common ontological background, their specific particularities are too diverse for data analytic purposes. This has been illustrated with the generation of multiple species-specific conceptual schemes where different genomic components have been identified as relevant, depending on the working context.

However, we have generated the CSG, which is a single, holistic conceptual schema that can be valid to work with every *eukaryotic* species because, even if they are unique and diverse, their genomes and how they behave are the same. This CSG works as a global, generic element from which conceptual views can be created in order to work with any specific species. We have presented the first steps towards the generation of the first version of the aforementioned CS by comparing two conceptual schemes that have been developed to work with different species: the CSHG for working with the human species, and the CSCG for working with citrus species.

We have identified what parts of the CSHG must be improved (i.e., extended, generalized, or specialized), and we have discussed the potential benefits of applying them. Eight genomic components that extend the CSHG have been identified: scaffolds, ortholog groups, introns, protein domains, the mRNA, and the structural components of the mRNA (5’ UTR, CDS, and 3’ UTR). The genotype frequency concept has been generalized from being related to a specific type of variation to be related to every type of variation. The concept of variation has been specialized so that ii is represented at three levels. It is represented at the general level, at the population level, and at the individual level.

The first working version of the generated CSG is a useful starting point that can be used in the human use case, in the citrus use case and, potentially, in more use cases of other species. Our plans for future works include the following: to work continuously towards the generation of additional versions that include new domain knowledge as it is obtained; to create two species-specific working conceptual views from the CSG (the human conceptual view of the CSG and the citrus view of the CSG); and to make a thorough study of domain concepts in order to explicitly separate purely biological data from technologically-oriented data.
